# Differential pricing: solidarity at times of financial crisis

**DOI:** 10.1186/1750-1172-9-S1-O28

**Published:** 2014-11-11

**Authors:** Flaminia Macchia

**Affiliations:** 1 EURORDIS

## 

The financial crisis which has hit Europe (and beyond) in 2008 has been described as a “health system shock” by the WHO which has urged EU Member States to ensure that the national healthcare systems would continue to promote universal access to health services by increasing efficiency in the health sector.

The challenge for Member States is to ensure access to innovative medicines for unmet medical needs, while at the same time meeting cost containment objectives and rewarding innovation.

The need for improving sustainability of EU health systems has led to thorough analysis of different pricing models in Europe which showed substantial support of a wide range of stakeholders (including research-based industry, patients and payers) in favour of a differential pricing system as a reimbursement policy measure for new and cost-intensive medicinal products, which is the case for most orphan medicinal products (OMPs).

One way to reduce the impact of OMPs on national healthcare budgets is to differentiate prices according to different levels of Gross Domestic Products (GDPs) and introduce some sense of proportionality between a country’s ability to pay and a fair level of reward for innovation.

The idea for this differential pricing system would therefore be to introduce a price differentiation not country by country but by “clusters of countries”: High, Medium and Low income countries, knowing that in Europe the GDP per capita varies from 1 to 6, with Luxembourg being the highest and Bulgaria the lowest.

In a first phase, differential pricing would be limited to medicinal products responding to high unmet medical need: a price would be agreed on the basis of a value-based pricing system, with the value being assessed with the EU Transparent Value Framework delivered by the MoCA (Mechanism of coordinated Access) process. Then variations by cluster would apply within a range from minus to plus 10%.

Two main obstacles to the introduction of a differential pricing system have been identified as being the External Reference Pricing and the practice of parallel imports.

The reflection process is still on-going at EU level on differential pricing as a way to address the three following main policy objectives: timely and equitable access to medicinal products for all patients, control of health expenditures linked to medicines and reward for innovation. There is an overall need for political support from Member States and industry.

